# The *phzA2-G2* Transcript Exhibits Direct RsmA-Mediated Activation in *Pseudomonas aeruginosa* M18

**DOI:** 10.1371/journal.pone.0089653

**Published:** 2014-02-24

**Authors:** Bin Ren, Huifeng Shen, Zhi John Lu, Haiming Liu, Yuquan Xu

**Affiliations:** 1 SKLMM of Life Sciences and Biotechnology School, Shanghai Jiao Tong University, Shanghai, People’s Republic of China; 2 MOE Key Laboratory of Bioinformatics, School of Life Sciences, Tsinghua University, Beijing, People’s Republic of China; Belgian Nuclear Research Centre SCK/CEN, Belgium

## Abstract

In bacteria, RNA-binding proteins of the RsmA/CsrA family act as post-transcriptional regulators that modulate translation initiation at target transcripts. The *Pseudomonas aeruginosa* genome contains two phenazine biosynthetic (*phz*) gene clusters, *phzA1-G1* (*phz1*) and *phzA2-G2* (*phz2*), each of which is responsible for phenazine-1-carboxylic acid (PCA) biosynthesis. In the present study, we show that RsmA exhibits differential gene regulation on two *phz* clusters in *P. aeruginosa* M18 at the post-transcriptional level. Based on the sequence analysis, four GGA motifs, the potential RsmA binding sites, are found on the 5′-untranslated region (UTR) of the *phz2* transcript. Studies with a series of *lacZ* reporter fusions, and gel mobility shift assays suggest that the third GGA motif (S3), located 21 nucleotides upstream of the Shine-Dalgarno (SD) sequence, is involved in direct RsmA-mediated activation of *phz2* expression. We therefore propose a novel model in which the binding of RsmA to the target S3 results in the destabilization of the stem-loop structure and the enhancement of ribosome access. This model could be fully supported by RNA structure prediction, free energy calculations, and nucleotide replacement studies. In contrast, various RsmA-mediated translation repression mechanisms have been identified in which RsmA binds near the SD sequence of target transcripts, thereby blocking ribosome access. Similarly, RsmA is shown to negatively regulate *phz1* expression. Our new findings suggest that the differential regulation exerted by RsmA on the two *phz* clusters may confer an advantage to *P. aeruginosa* over other pseudomonads containing only a single *phz* cluster in their genomes.

## Introduction

Over the last few decades, significant progress has been made in our understanding of the biosynthesis, genetics, and functional roles of various phenazines, particularly in pseudomonads [1]–[5]. It has been shown that phenazines control multiple bacterial behaviours in a variety of environments and play versatile roles as (i) signalling molecules to control gene expression [6], (ii) virulence factors in animals and humans [7], and (iii) antimicrobial agents against bacteria, fungi, and nematodes i.e. to protect plants [8]. Hence, it has been suggested that phenazines significantly contribute to the superior survivability of pseudomonads within competitive environments [9], [10], [11].

The *Pseudomonas aeruginosa* genome contains two *phz* gene clusters, *phzA1-G1* (*phz1*) and *phzA2-G2* (*phz2*), each of which encodes a full set of genes required to synthesise phenazine-1-carboxylic acid (PCA) [12], [13]. The two *phz* clusters in the genome are non-allelic and under the control of different upstream regulatory regions, in spite of the fact that the individual coding sequences of the two *phz* clusters are highly conserved in various *P. aeruginosa* genomes [12]–[15]. A regulatory feedback loop regulates the expression of both *phz* clusters, in which a small amount of PCA molecule produced by the *phz2* locus is able to activate expression of the *phz1* cluster, while on the other hand efficient expression of this cluster is largely blocked by its 5′-untranslated region (UTR) [16]. Recently, several genes involved in the regulation of phenazine biosynthesis and modification were investigated. In particular, the *phzH* gene in *P. aeruginosa* PAO1 was found to be involved in the conversion of PCA into phenazine-1-carboxamide (PCN) [13]. Moreover, the functions of the *phzM* and *phzS* genes that flank the *phz1* gene cluster in strain PAO1 are known to play critical roles during the conversion of PCA to pyocyanin (PYO) [13], [17], [18]. It has also been reported that *phzM* gene expression is temperature-dependent in a strain-specific manner [19]: as a result, more PCA is produced in the rhizosphere-originating strain of *P. aeruginosa* M18 at 28°C, whereas more PYO is produced, owing to the high expression of the *phzM* gene, in a *P. aeruginosa* clinical isolate PAO1 grown at 37°C. A ubiquitous RNA chaperone, Hfq, first discovered in 1968 as an *Escherichia coli* host factor required for bacteriophage Qβ replication and today known to bind a multitude of small regulatory RNAs (sRNAs) and as such to be involved in many regulatory activities in the cell [20], post-transcriptionally represses *phzM* expression and consequently reduces the conversion of PCA to PYO [21]. Hfq also positively controls expression of the *phz2* gene cluster to promote PCA biosynthesis through QscR-mediated transcriptional regulation at the promoter [21].

The GacS/GacA regulatory system consists of a sensor kinase (GacS) and a response regulator (GacA) [22]. The system is conserved in a range of Gram-negative bacteria and is also a key mediator of successful adaptation by microorganisms to changing environments. It was demonstrated that activated GacA also positively controls the transcription of sRNAs, such as RsmY and RsmZ [23], [24]. Over-expression of these sRNAs is thought to adjust the rate of translation initiation by sequestering RNA-binding proteins of the RsmA/CsrA family [25]–[30].

The RsmA protein, a CsrA homolog, is a symmetrical homodimer that contains two identical RNA-binding surfaces, each of which has a β-β-β-β-β-α secondary structure [31]. In target transcripts, repeated and appropriately spaced GGA motifs are essential for effective recognition by RsmA/CsrA protein. Members of the RsmA/CsrA family recognise specific binding sites in the 5′-untranslated leader of target transcripts and alter their translation and/or stability [32]–[36]. The RsmA regulon has recently been reported to include over 500 genes in *P. aeruginosa* PAK, of which about two-thirds are positively affected by an *rsmA* mutant and one-third are negatively affected. Moreover, a model is proposed in which RsmA acts directly as a negative translational regulator by competitively binding to the ribosome-binding region (SD sequence) of mRNA targets, and the positive effects of RsmA are achieved indirectly by RsmA-mediated interference with the translation of specific regulatory factors [34]. However, in *E. coli*, CsrA directly activates *flhDC* expression, which encodes for the regulator of flagellum biosynthesis, by protecting mRNA from RNase E-mediated cleavage [32], [36]. It remains unknown whether positive regulation by RsmA can occur directly at a post-transcriptional level in pseudomonads.

In our previous paper, we found that PCA production is negatively regulated by GacA in *P. aeruginosa* M18 [37]. This result is contradictory to a related report, in which the yield of PYO is positively regulated by GacA in *P. aeruginosa* PAO1 [38]. To date, the mechanisms allowing for a different control of GacA activity on *phz* gene expression remains elusive. In the present study, we sought to clarify the unusual features involved in RsmA regulation of the gene expression from the two *phz* loci in *P. aeruginosa* M18. Surprisingly, we found that expression of the two *phz* gene clusters was inversely regulated by direct RsmA-mediated activity, negatively on the *phz1* gene cluster and positively on *phz2* locus. Furthermore, we derived a novel model to explain the mechanism which might be involved in activation of the *phz2* transcript mediated directly by RsmA in *P. aeruginosa*.

## Results

### RsmA Differential Activity on Expression of Two *phz* Gene Clusters

**Figure 1:**
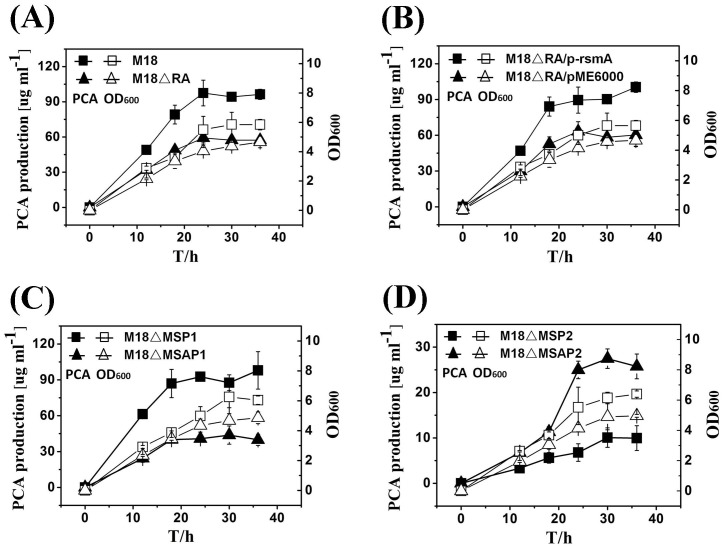
Effects of RsmA on PCA production and the growth of strain M18 and its mutants. Growth curves (open symbols) and PCA production (solid symbols) were determined for (A) the wild-type strain M18 (squares) and its *rsmA* mutant M18ΔRA (triangles), (B) M18ΔRA/p-rsmA (squares) and M18ΔRA/pME6000 (triangles), (C) triple-mutant M18ΔMSP1 (squares) and quadruple-mutant M18ΔMSAP1 (triangles), and (D) triple-mutant M18ΔMSP2 (squares) and quadruple-mutant M18ΔMSAP2 (triangles) in PPM broth at 28°C. M18ΔRA/pME6000 indicates *rsmA* mutant M18ΔRA harbouring an empty pME6000 plasmid. M18ΔRA/p-rsmA indicates *rsmA* mutant M18ΔRA harbouring recombinant pME6000 that expressed the *rsmA* gene. Values are the means ± standard deviations of triplicate cultures.

The general effects of RsmA on cellular growth and PCA production were investigated for the wild-type strain *P. aeruginosa* M18 and its *rsmA* deletion mutant M18ΔRA at 28°C during cultivating in PPM broth. These results showed that the growth of the mutant M18ΔRA was a little lower than that of wild-type strain M18, while the amount of PCA produced by the mutant M18ΔRA was only half of that produced by the wild-type strain M18, indicating that the total amount of PCA was positively regulated by RsmA at 28°C (Fig. 1A). These results were confirmed by a complementary experiment *in trans*, in which the decreased PCA production of the mutant M18ΔRA was restored to the wild-type level by introducing a plasmid-borne *rsmA* gene into the mutant M18ΔRA, which showed that the reduction of PCA production by M18ΔRA did not result from its lower growth rate (Fig. 1B).

Based on the previous reports, in *P. aeruginosa* PAO1 the two *phz* gene clusters (*phz1* and *phz2*) contribute to PCA biosynthesis, and three modification genes (*phzH*, *phzM*, and *phzS*) are involved in converting the synthesized PCA into various phenazine derivatives [13]. However, analysis of the complete genomic sequence of wild-type strain *P. aeruginosa* M18 has shown that this strain is actually defective in *phzH*, and the 302^nd^ codon of *phzH* is changed from CAG to TAG, resulting in nonsense mutation and gene inactivation of *phzH* (GenBank access no. CP002496, Gene number: PAM18_0053). To precisely determine the individual contribution of each *phz* cluster on PCA production without interference by the modification genes, we first created two triple-mutant strains, M18ΔMSP1 and M18ΔMSP2, in which the two *phzM* and *phzS* genes with either the *phz1* gene cluster or the *phz2* gene cluster were deleted in the wild-type strain M18. Then, two quadruple-mutant strains, M18ΔMSAP1 and M18ΔMSAP2, were constructed, in which *rsmA* was inactivated in the two respective triple-mutants, which aided investigating RsmA activity on each of the two *phz* clusters.

**Figure 2:**
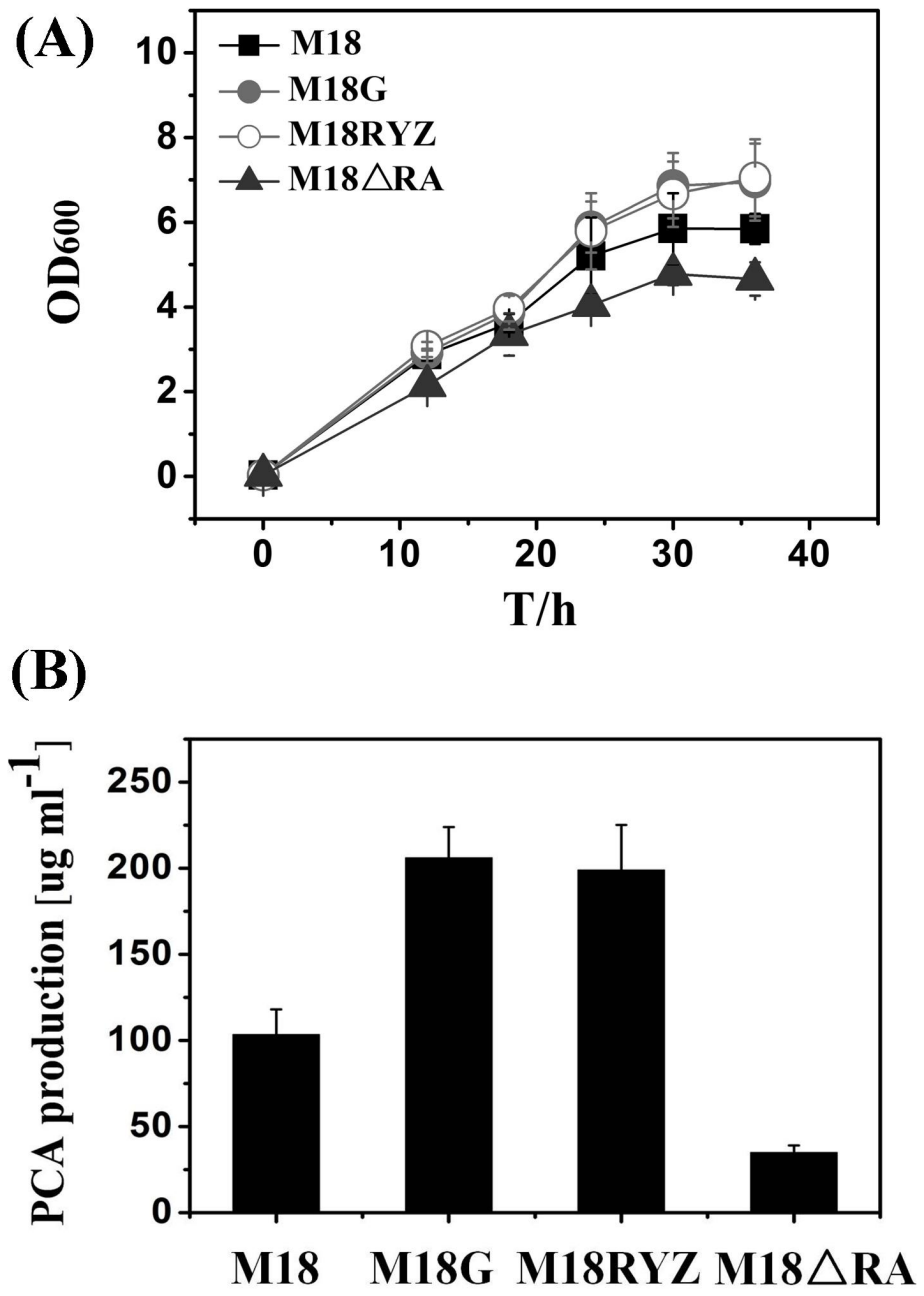
Effect of the Gac/Rsm system on the cell growth and PCA production. Cell growth assay (A) and PCA production (B) of the wild-type strain M18 and its derivative mutants were determined in PPM broth at 28°C. M18 (wild-type strain; solid squares), M18G (*gacA* mutant strain; solid circles), M18RYZ (*rsmY/Z* double mutant strain; open circles) and M18ΔRA (*rsmA* mutant strain; solid triangles).

We found that the quadruple-mutant M18ΔMSAP1 displayed a 70% reduction in PCA production as compared to the triple-mutant M18ΔMSP1, which indicated that expression of the *phz2* cluster was positively regulated by RsmA (Fig. 1C). PCA production by the quadruple-mutant M18ΔMSAP2 was three-fold higher than that of the triple-mutant M18ΔMSP2, showing that expression of the *phz1* cluster was negatively controlled by RsmA-mediated activity (Fig. 1D). Based on these results, we preliminarily concluded that RsmA in *P. aeruginosa* M18 exerts dual activities, i.e. either as a repressor of *phz1* gene cluster expression or as an activator of *phz2* gene cluster expression. In addition, PCA production at 28°C by the triple-mutant M18ΔMSP1 was eight-fold greater than that of the triple-mutant M18ΔMSP2 at the same temperature, which strongly indicates that expression of the *phz2* gene cluster would be the major contributor to PCA production in strain M18 at 28°C. As a consequence, it is likely that the effects of RsmA activation on total PCA production in M18, at 28°C, mainly resulted from gene control at the *phz2* locus. Furthermore, these conclusions possibly explained the PCA production at 28°C influenced by the other upstream components of Gac/Rsm regulatory pathway. In the *gacA* mutant strain M18G and/or *rsmY/Z* double mutant strain M18RYZ, RsmA could relieve the antagonism of sRNAs (RsmY and RsmZ) and trigger a stronger expression of *phz2* gene cluster, which yielded a higher PCA production than wild-type level (Fig. 2).

### Differential RsmA Regulation on the Two *phz* Gene Clusters Occurs at the Post-transcriptional Level

**Figure 3:**
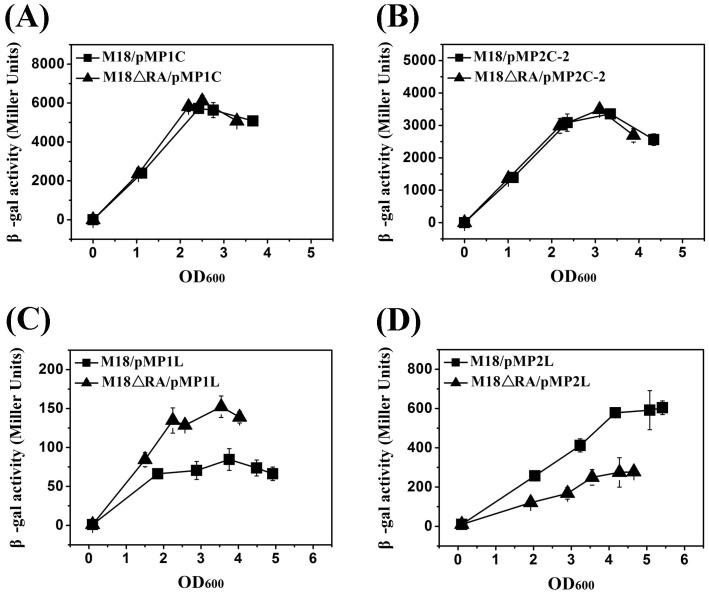
Activities of transcriptional and translational *lacZ* fusions for *phzA1* and *phzA2* in strain M18 and its *rsmA* mutant M18ΔRA. β-gal activities of the two transcriptional fusions of pMP1C (*phzA1-lacZ*) and pMP2C-2 (*phzA2-lacZ*) are shown in (A) and (B), and the activities of the two translational fusions of pMP1L (*phzA1′-’lacZ*) and pMP2L (*phzA2′-’lacZ*) are shown in (C) and (D) both in wild-type strain M18 (squares) and *rsmA* mutant M18ΔRA (triangles) in PPM broth at 28°C. Values are the means ± standard deviations of triplicate cultures.

To further investigate the results obtained above, we determined the β-galactosidase (β-gal) activities of transcriptional and translational *lacZ* fusions to *phzA1* and *phzA2* in both the wild-type strain M18 and its *rsmA* deletion mutant M18ΔRA. Based on their gene organisation, both *phzA1* and *phzA2* were encoded as the first genes in the two *phz* gene clusters (*phzA1-G1* and *phzA2-G2*). First, we determined the β-gal activities of the *phzA1*-*lacZ* transcriptional fusion (pMP1C) and the *phzA1*′-’*lacZ* translational fusion (pMP1L) [16] in both wild-type strain M18 and mutant M18ΔRA at 28°C. The β-gal activity of transcriptional fusion pMP1C was equivalent in these two strains, while β-gal activity of translational fusion pMP1L in the mutant M18ΔRA was two-fold higher than in the wild-type strain M18 (Fig. 3A and C).

We also investigated RsmA-mediated activation of *phz2* expression at transcriptional and translational levels. The newly constructed *phzA2*-*lacZ* transcriptional fusion (pMP2C-2) and the *phzA2′*-’*lacZ* translational fusion (pMP2L) [16] were delivered into the wild-type strain M18 and mutant M18ΔRA, and activities of the two fusions in these strains were determined at 28°C. These results showed that β-gal activity of the transcriptional fusion pMP2C-2 was the same in the two strains, while β-gal activity of the translational fusion pMP2L in the mutant M18ΔRA was one-third of that in the wild-type strain M18 (Fig. 3B and D). Overall, these results showed that RsmA-mediated differential control of the expression of the two *phz* gene clusters occurred for both at the post-transcriptional level.

### Identification and Sequence Analysis of Two *phz* Leader Regions

**Figure 4:**
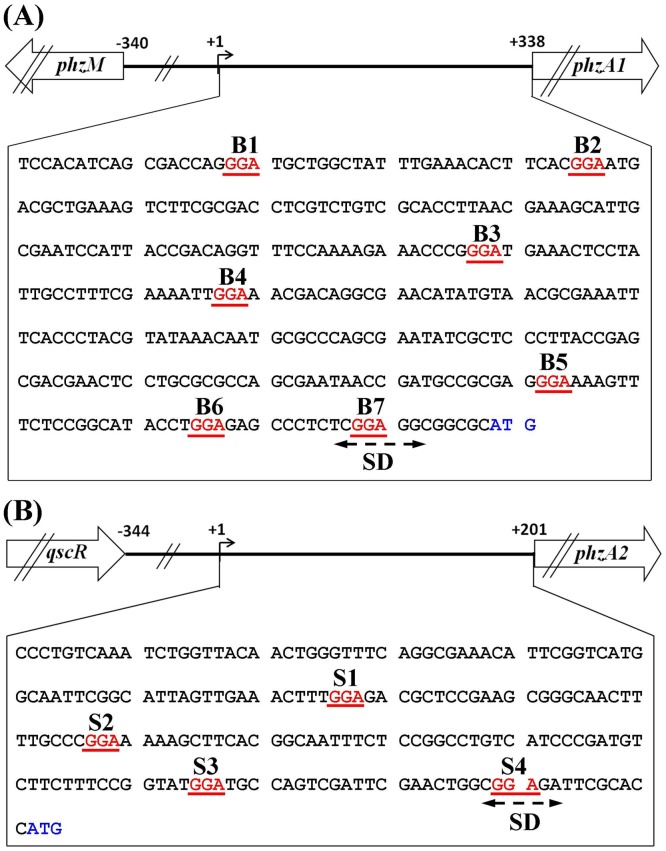
5′-UTR sequence of the two *phz* gene clusters. The 5′-untranslated nucleotide sequences between the transcriptional start site (TSS) and the translation start codon (ATG) in the *phzA1* (A) and *phzA2* (B) gene. B_1–7_ and S_1–4_ (GGA motifs): potential RsmA binding sites.

The transcription start sites (TSS, designated as +1) of the two *phz* gene clusters were explored in our previous study [16]. Based on the sequence analysis, we found seven 5′-GGA-3′ motifs (designated B1–B7) within the 5′-UTR of the *phz1* transcript and a potential 5′-CUCGGAGG-3′ RsmA binding site that overlapped with the predicted SD sequence (Fig. 4A). In the previous reports, various RsmA/CsrA-mediated translation repression mechanisms have been identified in which RsmA/CsrA binds near the SD sequence of target transcripts, thereby blocking ribosome access [35], [39]. As a result, we speculated that RsmA binding to the *phz1* transcript favoured hindering ribosome access and translation initiation, possibly explaining the RsmA-mediated repression of *phz1* translation.

In contrast, the four potential RsmA target motifs 5′-GGA-3′ (designated S1, S2, S3, and S4) were found within the 5′-UTR of *phz2* transcript in *P. aeruginosa* M18 (Fig. 4B), although the mechanism of RsmA-mediated activation was unknown. In *E. coli*, a CsrA-mediated activation mechanism was identified in which CsrA increased *flhDC* expression by stabilising the transcript [32], [36]. To determine whether *phz2* mRNA stability was affected by RsmA *in vivo*, we investigated the *phz2* transcript levels by qRT-PCR in the wild-type strain M18 and mutant M18ΔRA using the housekeeping gene *rpoD* as a control. During the exponential and stationary phases, the normalised *phz2* transcript level in the mutant M18ΔRA was similar to that in the wild-type strain M18, which demonstrated that the absence of RsmA did not affect *phz2* transcript stability significantly (Table 1). Therefore, this result eliminated a possible model in which RsmA activated *phz2* expression by stabilising its mRNA.

**Table 1:** Relative *phzA2* transcript levels in the M18 and M18ΔRA strains during the exponential and stationary phases as assessed by qRT-PCR.

Strains	Exponential phase				Stationary phase			
	C_T, *rpoD*_ a	C_T, *phzA2*_ a	ΔC_T_ b	2^–ΔΔC^T^c^	C_T, *rpoD*_ a	C_T, *phzA2*_ a	ΔC_T_ b	2^–ΔΔC^T^c^
M18	19.47±0.06	19.47±0.15	0	**1.00**	18.81±0.04	21.18±0.02	2.36	**1.00**
M18ΔRA	19.48±0.03	18.90±0.04	−0.58	**1.49**	20.05±0.04	21.72±0.15	1.68	**1.61**

a Values were determined during the exponential phase (OD_600_ between 1.5 and 2.0) and the stationary phase (OD_600_ between 5.0 and 5.5). Values are means ± standard deviations of three independent experiments.

b ΔC_T_ = mean C_T,*phzA2*_– mean C_T,*rpoD*_.

c 2^–ΔΔC^T, Normalised amount of cDNA from the *phzA2* gene in the mutant M18ΔRA relative to that in the wild-type strain M18; ΔΔC_T_ = mean ΔC_T_ – meanΔC_T,M18_.

### Determination of RsmA Binding Site of the *phz2* Transcript

**Figure 5:**
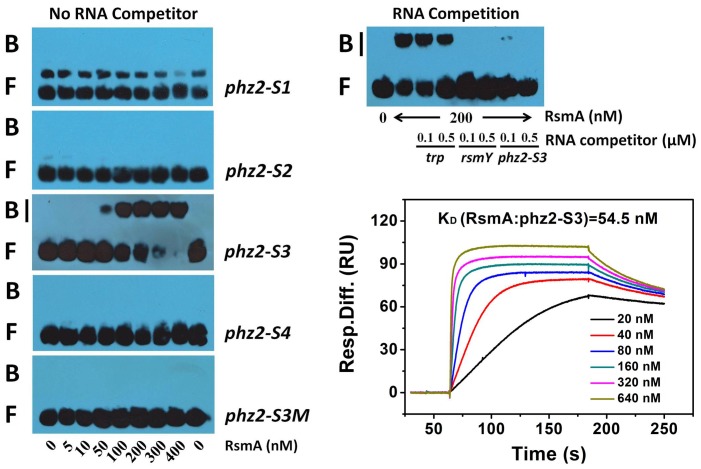
Gel mobility shift assays for RsmA protein direct binding to the 5′-UTR of the *phz2* transcript. 3′ end-labeled WT *phz2-S1*, *phz2-S2*, *phz2-S3*, *phz2-S4* and mutant *phz2-S3M* transcripts (1 nM) were incubated with RsmA (concentration indicated at the bottom of each lane). Positions of bound (B) and free (F) *phz2* leader RNA are marked. For an RNA competition assay, labeled *phz2-S3* RNA was incubated with RsmA ±100- or 500-fold excess of non-specific (*trp*) and specific (*rsmY* and *phz2-S3*) competitor RNA. The concentrations of RsmA and competitor RNA were shown at the bottom of the corresponding lanes. The equilibrium binding constant (K_D_) of RsmA binding to WT *phz2-S3* was calculated to be 54.5 nM using surface plasmon resonance assays.

The results obtained above compelled us to explore how the RsmA protein might interact with the *phz2* transcript. To determine the actual RsmA binding site, we synthesised four different parts of a biotin-labelled leader for *phz2* mRNA and purified histidine-tagged RsmA to conduct gel mobility shift assays (Fig. 5). The four synthesised RNA molecules (designated *phz2-S1*, *phz2-S2*, *phz2-S3*, and *phz2-S4*) represented different regions that covered the S1, S2, S3, and S4 motifs, respectively. RsmA had specific affinity for *phz2-S3* RNA and this complex was formed at an RsmA concentration of >50 nM, whereas RsmA did not bind to *phz2-S1*, *phz2-S2*, and *phz2-S4* RNAs. To confirm the efficiency of RsmA binding to this target, we synthesised an altered *phz2-S3* RNA (*phz2-S3M*) in which the 5′-GGA-3′ sequence was replaced by 5′-UUC-3′, and this substitution resulted in the loss of RsmA–RNA complexes. Further, the specificity of the RsmA-*phz2-S3* RNA interaction was investigated using competition experiments with specific (*rsmY* and *phz2-S3*) and non-specific (*trp*) competitors. Unlabelled *rsmY* and *phz2-S3* were effective competitors for RsmA-*phz2-S3* RNA interactions, whereas only minimal competition was observed with RNA derived from *trp*, which demonstrated that RsmA was bound specifically to *phz2-S3* RNA. To estimate the affinity of RsmA to the *phz2-S3* RNA, equilibrium analyses of the interaction were performed using surface plasmon resonance (SPR). The quantification of RsmA-*phz2-S3* interaction revealed an equilibrium binding constant (K_D_) value of 54.5 nM (Fig. 5). In all, these results suggested that the S3 motif was indeed the direct target of RsmA.

### RsmA Contributes to Activating *phz2* Expression Via the S3 Motif

**Figure 6:**
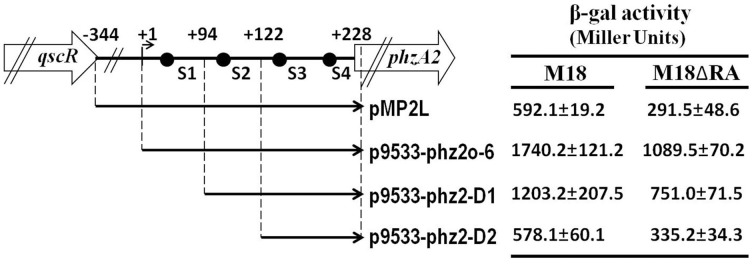
Positive regulation of *phz2* expression by RsmA and identification of a target regulatory sequence within the *phz2* operon leader. Construction map for a series of translational and post-transcriptional *lacZ* fusions for *phzA2* and the β-gal activities of the four fusions were determined in wild-type strain M18 and mutant M18ΔRA in PPM broth at 28°C. The possible regulatory site of RsmA on the activating region was located at +122 to +228 nt downstream of the *phzA2* TSS. Values are the means ± standard deviations of triplicate cultures.

To further define the target regulatory elements of RsmA within the 5′UTR of the *phz2* gene cluster, we constructed a translational fusion plasmid pMP2L (containing sequences from bp −344 to +228) and three post-transcriptional fusion plasmids, p9533-phz2o-6 (from bp +1 to +228), p9533-phz2-D1 (from bp +94 to +228), and p9533-phz2-D2 (from bp +122 to +228). The four fusions were delivered into the wild-type strain M18 and mutant M18ΔRA and their *lacZ* expressions were measured and compared at 28°C in PPM broth (Fig. 6). Deletion of *rsmA* caused a significant decrease in LacZ activity expressed by a translational fusion plasmid pMP2L and three post-transcriptional fusion plasmids. Based on these results, we concluded that the non-transcriptional region (from bp −344 to +1) and partial 5′ leader region (from bp +1 to +122) was not essential for RsmA activation, and the key regulatory fragment that mediated positive regulation by RsmA was located from sites +122 to +228 downstream of the *phz2* transcript TSS, in which included the RsmA binding site (S3). Taken all results together, it appears that the S3 motif located 21 nucleotides upstream of the SD sequence is involved in the direct RsmA-mediated activation of *phz2* expression.

### A Proposed model for RsmA Direct Activation of *phz2* Translation

**Figure 7:**
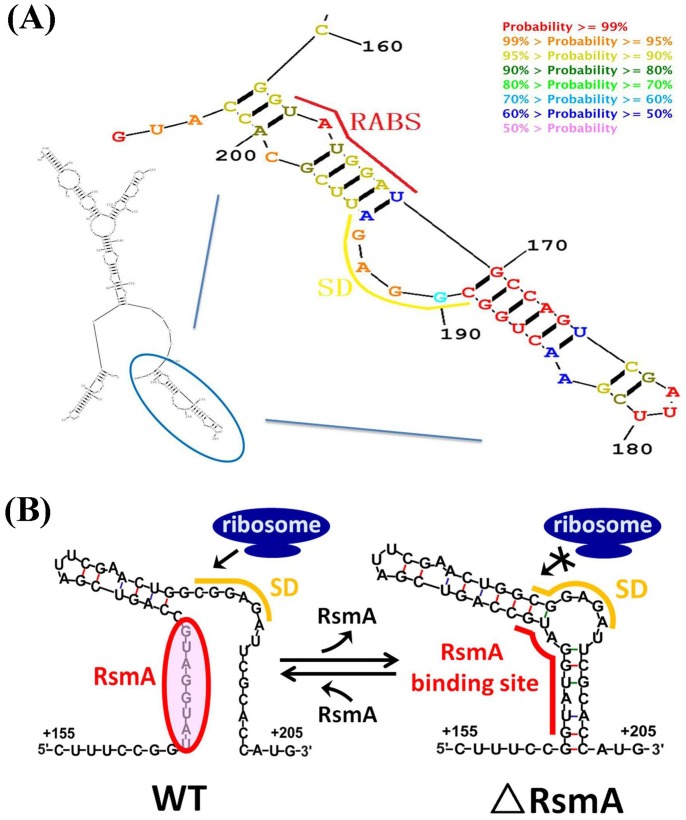
Structure prediction of the *phz2* transcript and putative model of RsmA mediated activation of *phz2* translation. (A) A predicted 5′-UTR secondary structure from +160 to +205 nucleotides of the *phz2* transcript generated by *RNA structure.* (B) A putative direct activation model of the *phz2* transcript mediated by RsmA in *P. aeruginosa* M18. In the *rsmA*-deleted mutant (right), base-paired nucleotides between the SD with its flanking sequence and the RABS resulted in a relatively stable stem-loop structure, which prevented ribosome access and translation initiation. However, in the WT strain (left), the loose stem-loop structure caused by RsmA binding resulted in easy access to the ribosome and translation activation of the *phz2* transcript. RABS: RsmA binding site.

We predicted the optimal secondary structure of the *phz2* leader spanning region using *RNA Structure* [40] (Fig. 7A). The region between nucleotides +160 to +205 was found to most likely form a large stem-loop structure in which the SD sequence was trapped. We postulated that RsmA binding to the target S3 motif (RABS) would result in destabilising this stem-loop structure and might promote access of the 30S ribosome subunit on the transcript, thereby allowing translation initiation (Fig. 7B).

**Figure 8:**
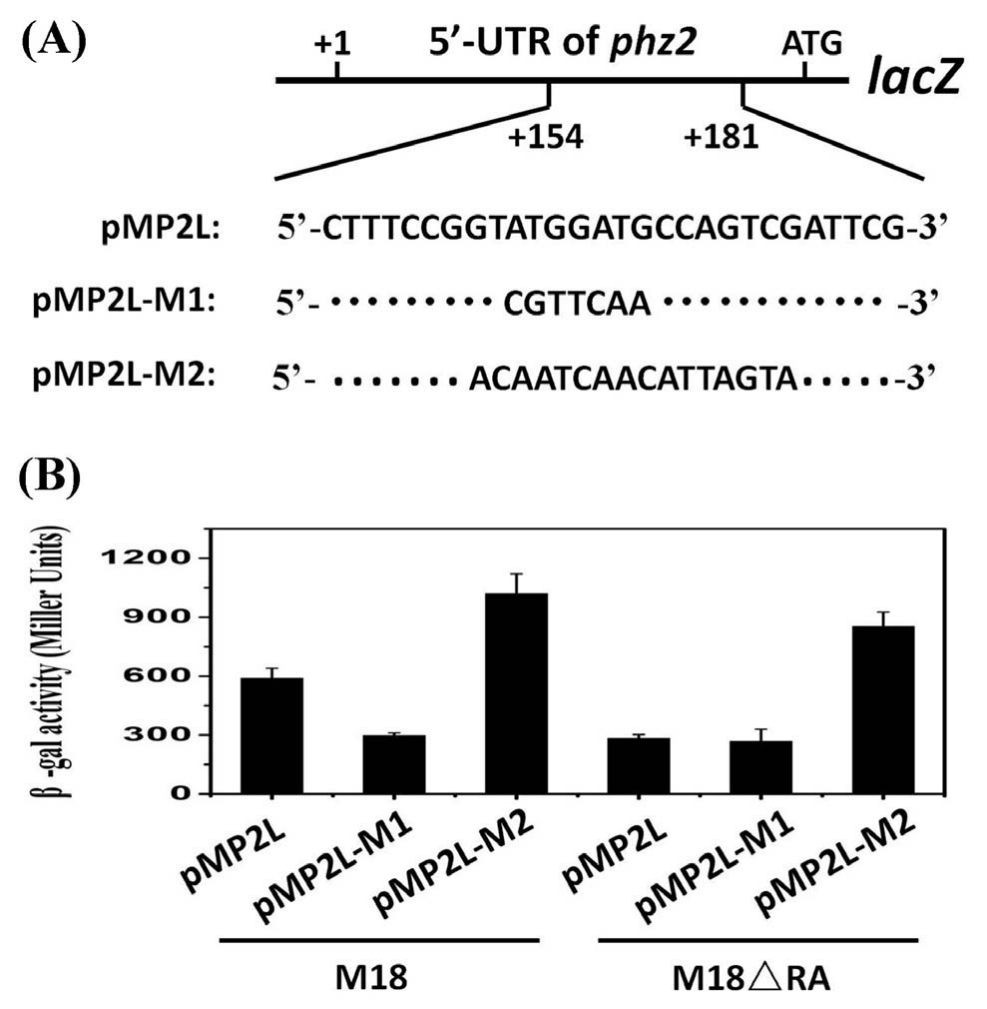
Functional analysis of the putative RsmA-binding region and its flanking sequences of *phz2* transcripts. (A) Construction of a WT *phzA2′-‘lacZ* translational fusion and two mutant fusions for pMP2L-M1 and pMP2L-M2 in which the RsmA-binding site (RABS) and both RABS along with its flanking sequences for the *phz2* leader region were replaced, respectively. (B) β-gal activities of the three fusions were determined in strain M18 and mutant M18ΔRA in PPM broth at 28°C. Symbols: pMP2L, translational fusion of WT *phz2* cluster; pMP2L-M1 fusion, containing a substitution of the RsmA binding site; pMP2L-M2 fusion, containing a substitution of the paired-base region. Values are the means ± standard deviations of triplicate cultures.

**Figure 9:**
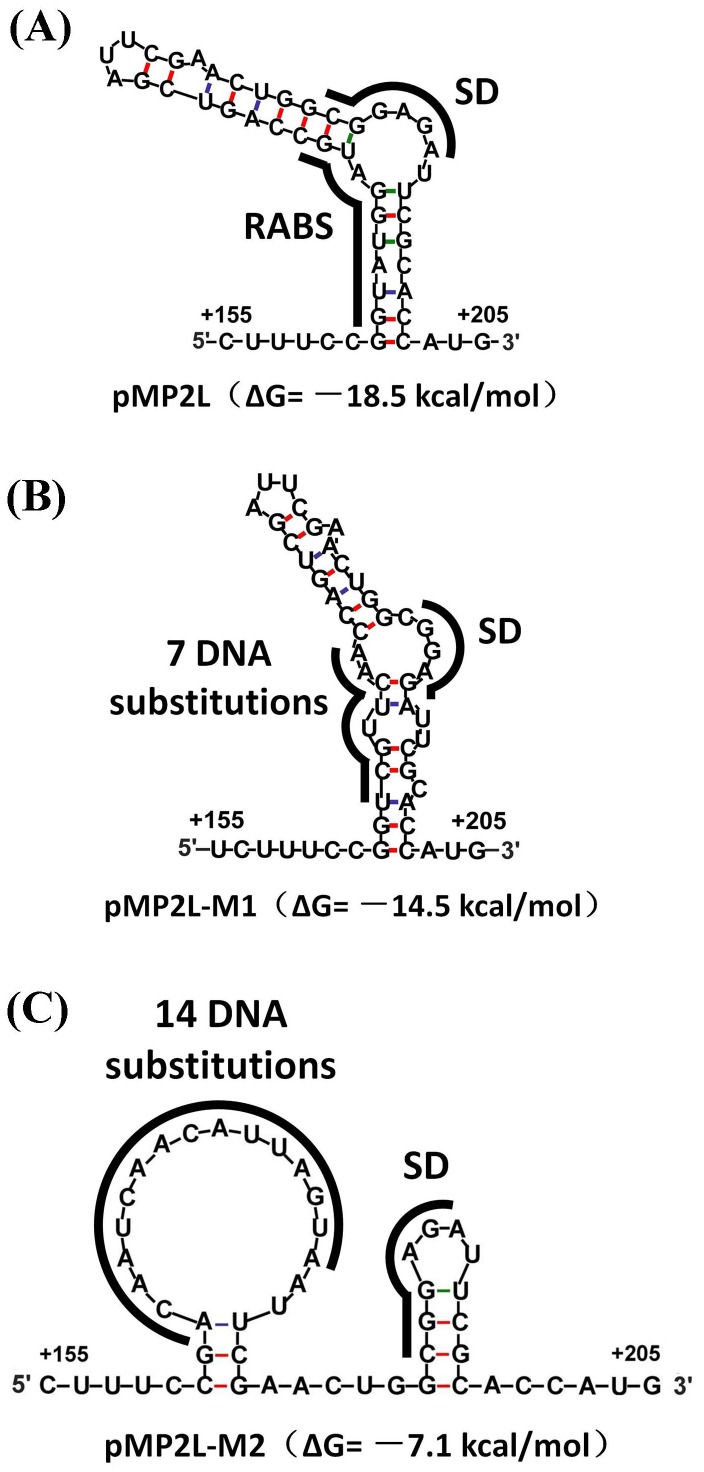
Secondary structures and free energies of WT and mutant *phz2* leader regions. The RNA structures of the *phz2* upstream region from nt +160 to +205 in the three plasmids of pMP2L (A), pMP2L-M1 (B), pMP2L-M2 (C) were predicted by M-fold and their folding free energies were -18.5 kcal/mol, -14.5 kcal/mol, and-7.1 kcal/mol, respectively. RABS: RsmA binding site.

To further explore whether this stem-loop structure is required for RsmA binding and RsmA-mediated translation activation, two mutants, pMP2L-M1 and pMP2L-M2, were constructed, in which the RsmA target motif and the paired-base regions of the plasmid pMP2L were substituted, respectively (Fig. 8A). The mutant pMP2L-M1 contained seven consecutive DNA substitutions (A163C, T164G, G165T, G166T, A167C, T168A, and G169A), in which RsmA could not destabilize the stem-loop structure due to the absence of its target motif and the SD sequence was blocked. In contrast, the mutant pMP2L-M2, harbouring 14 DNA substitutions (G161A, T162C, T164A, G165T, G166C, T168A, G169C, C170A, C171T, A172T, G173A, T174G, C175T, and G176A) was predicted to prevent base-pairing and release the SD sequence in the presence or absence of RsmA protein. Using the RNA fold web server [41], the folding free energy of the *phz2* upstream region from nt +160 to +205 in the plasmid pMP2L was determined to be −18.5 kcal/mol, which was slightly lower than that in plasmid pMP2L-M1 (−14.5 kcal/mol) while it was significantly lower than that in plasmid pMP2L-M2 (−7.1 kcal/mol) (Fig. 9). The strains M18 and M18ΔRA transformed with pMP2L and its two mutants (pMP2L-M1 and pMP2L-M2) were assayed for β-galactosidase (LacZ) activity in PPM broth (Fig. 8B). In the mutant M18ΔRA, RsmA-mediated activation was lost and the translation efficiency of three plasmids was reflected by the stability of RNA structure. It was found that two plasmids of pMP2L and pMP2L-M1 exhibited relatively lower and similar LacZ activities while pMP2L-M2 showed a three-fold higher one. These results suggested that the structures in pMP2L and pMP2L-M1 shared a similar stability and that they were much more stable than that in pMP2L-M2, which corroborated with the free energy calculation of the RNA structures for the three plasmid constructs. In the wild-type strain M18, a two-fold increase in the *lacZ* expression from pMP2L was observed compared with that in mutant M18ΔRA, which indicated that the binding of RsmA on the target motif likely resulted in the destabilization of stem-loop structure and the enhancement of ribosome access. However, the fusion of pMP2L-M1 exhibited a similarly lower LacZ activity in the two strains (M18 and M18ΔRA) due to the substitution of RsmA specific motif. This result further suggested that the predicted RsmA target motif was essential for an efficient *phz2* translation. In contrast, fusion pMP2L-M2 exhibited a three-fold higher translation efficiency owing to the fact that the paired bases of the stem-loop structure were almost all substituted and hence the SD sequence was practically made free, i.e. exposed. In regard to the reporter fusion studies, RNA structure prediction, and gel mobility shift assays, all of the presented results support this activation model while more studies will be needed to clarify the related molecular mechanisms.

## Discussion

To our knowledge this is the first study in *P. aeruginosa* M18 that investigates the effect of RsmA activity on the expression control of the strain’s two *phz* gene clusters, and that determines the PCA production originating from each *phz* gene cluster without having to account for the possible interference by PCA modification genes. Interestingly, we found that expression of the two *phz* gene clusters was inversely regulated by direct RsmA-mediated activity, i.e., negatively on the *phz1* gene cluster and positively on *phz2* cluster. Our previous statement that PCA biosynthesis was not affected by RsmA in KMB broth [42] was premature because some important details were neglected. First, the yield of PCA produced from KMB broth was low to very low, which led us to overlook the difference of PCA production between the wild-type strain M18 and *rsmA*-inactivated mutant M18R^-^ (renamed as M18ΔRA in the present study), notwithstanding the fact that the mutant M18R^-^ displayed a 50% reduction in PCA production as compared to the PCA production in the wild-type strain M18. However, PCA production was significantly increased when PPM broth was adopted in the present study, and RsmA-mediated positive regulation on PCA biosynthesis in PPM became much more obvious than in KMB. Second, in the previous report, contribution of the two *phz* gene clusters on PCA production was not individually investigated and the construction of *phzA*‘-’*lacZ* translational fusion pMEZA [42] turned out to be faulty, hence our earlier misinterpretation of results and inaccurate conclusions.

Members of the sRNA binding protein RsmA/CsrA family modulate the efficiency of translation at target transcripts. Various RsmA/CsrA-mediated translation repression mechanisms have been identified, in which RsmA/CsrA binds near the SD sequence of target transcripts, thereby blocking the access of the ribosome [35], [39]. Similarly, in the present study, RsmA was shown to negatively regulate *phz1* expression. Despite evidence for the activation of several genes in *E. coli* [32], [43], the best example of CsrA-mediated activation is the increased stabilisation of *flhDC* mRNA. It has been found that CsrA activates *flhDC* expression by binding to the extreme 5′ end of this mRNA and inhibiting 5′ end-dependent cleavage by RNase E [36]. Moreover, Patterson-Fortin *et al*. suggest that CsrA post-transcriptionally activates *moaA* expression by altering the conformation of *moaA* mRNA to facilitate ribosome recruitment, and that CsrA binding to *moaA* transcript is necessary but not sufficient to activate *moaA* translation [44]. In the present study, studies with a series of *lacZ* reporter fusions, RNA structure prediction, free energy calculations, and gel mobility shift assays suggest that the third GGA motif (S3) located 21 nucleotides upstream of the SD sequence is involved in direct RsmA-mediated activation on *phz2* expression. Based on reporter fusion studies and qRT-PCR, these findings now suggest that RsmA mediates *phz2* translational activation without significantly altering *phz2* mRNA levels, and that RsmA is not involved in *phz2* activation by stabilising its mRNA. We propose a novel model in which RsmA post-transcriptionally activates *phz2* expression by destabilising a stem-loop structure and enhancing ribosome access in *P. aeruginosa* M18. Although all of the present results support this activation model, we realise that more studies are needed as to elucidate all underlying molecular mechanisms.

As recently reported, sRNAs that are involved in translational control can be divided into two major groups. sRNAs of the first class are considered to adjust the rate of translational initiation by sequestering proteins of the RsmA/CsrA family [27], [29]. In contrast, sRNAs of the second class act by base-paring with a target mRNA, usually assisted by the RNA chaperone Hfq [45]. Furthermore, Hfq or Hfq-mediated sRNAs reportedly play opposite roles on target transcripts. In our previous report [21], a typical secondary structure in which two stem-loops were linked by a short single-stranded AU-rich spacer could be considered as the target of Hfq in the mRNA leaders of negative Hfq regulons, including *pltR*, *phzM* and *qscR*. Hfq represses target mRNAs presumably by stabilising the inhibitory structure, thereby blocking ribosome access and anti-sense degradation with sRNAs. In contrast, Hfq in conjunction with sRNA has also been reported to directly activate an mRNA target by opening up the inhibitory structure or by facilitating a stable sRNA–mRNA complex [46]. An example of this is found with the translational activation of *rpoS* by Hfq and its related sRNAs (DsrA, RprA and ArcZ) [47]. Thus, whether one transcript can be negatively or positively regulated by Hfq depends on the specific secondary structure of the target mRNA. The dual regulatory pattern of RsmA on the expression of the two *phz* transcripts in the present study again demonstrates the crucial function of the specific sequence and structure of the 5′-UTR of a target mRNA.

The capability to sense environmental conditions and respond successfully is essential for the survival of organisms, particularly single-celled prokaryotes. The differential expression and regulation of redundant genes could relax selection pressure and allow for diversification [48]. Based on a recent report [49], the two redundant *phz* gene clusters in the adaptable bacterium *P. aeruginosa* PA14 exhibited environment-dependent expression. In this study the different contributions of the two *phz* gene clusters to phenazine biosynthesis were compared and evaluated in colonies and liquid cultures. The results indicated that the non-homologous promoter regions of the two *phz* gene clusters allowed for condition-dependent regulation of PCA biosynthesis and that oxygen acted as an environmental factor to facilitate *P. aeruginosa* adaption in different environments [49]. Furthermore, Wurtzel *et al*. [50] used RNA-seq to examine the temperature-dependent expression of the two *phz* gene clusters in *P. aeruginosa* PA14, which was grown at 28°C and 37°C. That study indicated that levels of transcripts from both of the *phz* clusters were elevated at 37°C, although the temperature effect was less pronounced for the *phz2* cluster. In the present study, PCA production from the two triple-mutant strains (M18ΔMSP1 and M18ΔMSP2) and two quadruple-mutant strains (M18ΔMSAP1 and M18ΔMSAP2) was further measured at 37°C (Fig. S1), and compared with the results obtained above at 28°C (Fig. 1C and D). We found that RsmA acted as an activator on the *phz2* gene cluster and as a repressor on the *phz1* cluster either at 28°C or 37°C, and the expression of two *phz* gene clusters exhibited differential thermoregulation, indicating that temperature and RsmA have their own specific regulatory mechanism to PCA biosynthesis and that regulation of the two *phz* transcripts might be more intricate than we originally thought. As explained in the introduction, GacA mediates differential regulation on the phenazine production at 28°C and 37°C [37], [38]. The phenomena may in fact be due to a condition-dependent expression of the two *phz* gene clusters in diverse environments.

All of our results suggest that RsmA-mediated differential regulation on the expression of two temperature-sensitive *phz* gene clusters may confer an advantage to *P. aeruginosa* over other pseudomonads that have only a single *phz* cluster in their genomes, and thus may help *P. aeruginosa* to survive and establish itself in diverse environments. Furthermore, the present results confirm that the relationship between the two *phz* gene cluster expressions is intricate and complex, as more than 32 genes were found to be involved in the regulation of the *phz1* gene cluster [51] while the factors involved in the regulatory expression of the *phz2* gene cluster remain largely unknown. The latest discovery of multiple non-identical copies of RsmA (RsmF and RsmN) may add a further complexity to post-transcriptional regulation on the expression of two *phz* gene clusters in *P. aeruginosa* [52], [53]. Clearly, more research is needed to elucidate the nature and action of these known and unknown factors so that the reciprocal influence of the expression of both *phz* gene clusters may be better understood.

## Materials and Methods

### Strains, Plasmids, Primers and Culture Conditions

All strains used in the present study were derived from *P. aeruginosa* M18. The bacterial strains and plasmids and the oligonucleotide primers used in the present study are listed in Tables S1 and S2, respectively. *P. aeruginosa* M18 and its deratives were incubated in King’s medium B (KMB) [54] for growth, and pigment-production medium (PPM) [55] for secondary metabolite PCA production. *E. coli* were routinely grown at 37°C in Luria-Bertani medium (LB) [56]. Antibiotics were added at the following concentrations: gentamicin (Gm) at 50 µg/mL, kanamycin (Km) at 50 µg/mL, spectinomycin (Sp) at 100 µg/mL, and tetracycline (Tc) at 125 µg/mL for *P. aeruginosa* strains and Km at 50 µg/mL, Gm at 25 µg/mL and Tc at 15 µg/mL for *E*. *coli*.

### DNA Manipulation and Cloning

All molecular biological procedures were done using standard methods. *Taq-*, LA *Taq-*, and *Pfu* DNA polymerases, restriction endonucleases, DNA ligase, RNA reverse transcriptase, DNA molecular mass markers, and associated products were used as recommended by the respective manufacturers (Fermentas-Thermo Fisher Scientific, Bejing, CN; TaKaRa, Dalian, CN; MBI, Ningbo City, CN). Genomic DNA was extracted using an EZ spin column genomic DNA isolation kit (Bio Basic Inc., Toronto, CAN). Plasmid DNA was prepared using a TaKaRa miniBEST plasmid purification kit, version 2.0. Restriction fragments were purified with an AxyPrep DNA gel extraction kit. DNA was sequenced and synthesised by Beijing HuaDa Genomics Institute and Invitrogen Biotechnology Corporation.

### Bioinformatics Analysis of the 5′-UTRs of Two *phz* Transcripts

The 5′-UTRs of *phz1* and *phz2* transcript sequence were aligned with the *P. aeruginosa* M18 genome using NCBI BLAST. The consensus sequence motifs of RsmA directly mediated on these two *phz* transcripts were identified by MEME [57]. Then, the secondary structures of *phz2* RNA were predicted with *RNA structure* [40] and M-fold [58].

### Construction of *rsmYrsmZ* Inactivated Double Mutant M18RYZ

Four primer pairs, PY1F-PY1R and PY2F-PY2R, and PZ1F-PZ1R and PZ2F-PZ2R (Table S2), were designed according to the sequences of two sRNAs genes (*rsmY* and *rsmZ*) and their flanking regions in *P. aeruginosa* M18, respectively. PCR products of *rsmY* gene were digested with *Sac*I/*Bam*HI (836 bp upstream fragment) and *Bam*HI/*Hin*dIII (1005 bp downstream fragment), and ligated to generate an 1841-bp fragment with a 72-bp deletion but leaving its promoter and terminator. The ligation product of deleted *rsmY* was cloned into the *Sac*I/*Hin*dIII-cut vector pEX18TC and an 825-bp fragment containing the Gm^r^ cassette from vector pUCGm was inserted into the *Bam*HI trimmed site, resulting in the recombinant construct pEX-Y1Y2-Gm (Table S1). Similarly, the up- and downstream fragments of *rsmZ* gene were digested with *Hin*dIII/*Xba*I and *Xba*I/*Kpn*I, and ligated to generate an 1811-bp product, which was cloned into the *Hin*dIII/*Kpn*I-cut vector pEX18TC. At the same time, a 1250-bp fragment containing the Km^r^ cassette from vector pUCKm was inserted into the *Xba*I trimmed site, resulting in the secondary recombinant construct pEX-Z1Z2-Km (Table S1). The two resulting plasmids, pEX-Y1Y2-Gm and pEX-Z1Z2-Km, were transformed from *E. coli* SM10 into *P. aeruginosa* M18 by biparental mating. The clones in which a double crossover had occurred were selected on plates containing Sp (100 µg/mL), Gm (50 µg/mL), Km (50 µg/mL) and 15% (w/v) sucrose. The *rsmYrsmZ* double mutant, designated as M18RYZ, was confirmed with PCR and sequencing.

### Construction of an *rsmA* Deletion Mutant and Its Complementation

To inactivate the *rsmA* gene, chromosomal genomic DNA was extracted and purified from *P. aeruginosa* M18. The up- and downstream PCR products that flanked the *rsmA* open reading frame (ORF) were amplified with LA *Taq* DNA polymerase and two primer pairs, PA1F and PA1R, and PA2F and PA2R, respectively (Table S2), and ligated to a 1052-bp fragment with a 400-bp deletion of the *rsmA* gene. This ligation product was cloned into *Eco*RI/*Hin*dIII-cut vector pK18mobsacB to generate the recombinant plasmid pK18-A1A2 (Table S1). This construct was transformed into *E. coli* strain S17 and subsequently mobilised into strain M18 by conjugation. Transconjugants were selected on LB medium supplemented with Sp (100 µg/mL) and Km (50 µg/mL). Positive colonies were plated on LB medium containing 5% (w/v) sucrose and Sp (100 µg/mL) to select for resolution of the construct by a second cross-over event. The resulting mutant M18ΔRA containing the *rsmA* in-frame deletion was confirmed by direct sequencing.

To make a complementation of the *rsmA* mutant, a 377-bp PCR fragment containing the *rsmA* encoding region along with its promoter region was amplified by the primers PACF/PACR (Table S2) from the genomic DNA of *P. aeruginosa* M18, recovered from agarose gel electrophoresis, purified and digested with *Hin*dIII/*Eco*RI, and then inserted into vector pME6000 to obtain p*-rsmA* (Table S1). The empty vector pME6000 and recombinant p*-rsmA* were transferred into mutant M18ΔRA.

### Construction of Two Quadruple-mutant Strains: M18ΔMSAP1 and M18ΔMSAP2

Four primer pairs, PP1F-PP1R and PP2F-PP2R, and PP3F-PP3R and PP4F-PP4R (Table S2), were designed based on the sequences of the two *phz* gene clusters in *P. aeruginosa* M18, respectively. The PCR products of the *phz1* gene cluster were digested with *Bam*HI/*Sac*I (1022 bp upstream fragment) and *Sac*I/*Xba*I (882 bp downstream fragment), and ligated to generate a 1904-bp fragment with a 6238-bp deletion, but leaving its promoter and terminator. This ligation product was cloned into *Bam*HI/*Xba*I-cut exchange vector pK18mobsacB, which resulted in the recombinant construct pK18-P1P2 (Table S1). Similarly, the up- and downstream fragments of the *phz2* gene cluster were digested with *Bam*HI/*Sac*I and *Sac*I/*Xba*I and ligated to generate a 2292-bp product, which was cloned into *Bam*HI/*Xba*I-cut pK18mobsacB and resulted in the secondary recombinant construct pK18-P3P4 (Table S1). The two resulting plasmids, pK18-P1P2 and pK18-P3P4, were transformed into *E. coli* strain S17 and subsequently mobilised into *P. aeruginosa* M18ΔMS (unpublished data) by conjugation. Two triple-mutants, designated M18ΔMSP1 and M18ΔMSP2, were generated using the methods described above. Subsequently, two quadruple-mutants of M18ΔMSAP1 and M18ΔMSAP2 were further constructed in the triple-mutants M18ΔMSP1 and M18ΔMSP2.

### Construction of a Transcriptional Fusion of the *phz2* Gene Cluster

A 210-bp PCR fragment from –209 to +1 (relative to the first transcriptional start point) containing the *phzA2-G2* promoter region was amplified from strain M18 genomic DNA using the primer pairs P2CF/P2CR (Table S2). This product was cloned into *Eco*RI/*Pst*I digested vector pME6522 to form the transcriptional fusion plasmid pMP2C-2 (Table S1), which was confirmed by direct sequencing.

### Replacements of Target RsmA and Its Flanking Sequence in the *phz2* Leader Region

Two *lacZ* reporter plasmids for which the RsmA target motif and its flanking sequences in the *phz2* leader region were replaced, respectively, were constructed to assess RsmA positive regulation on *phz2* expression. The first replaced fragment (–344 to +228), which contained a 7-nucleotide substitution (5′-ATGGATG-3′ to 5′-CGTTCAA-3′) in the RsmA target motif, was synthesised and cloned into an *Eco*RI/*Pst*I-cut pME6015 vector, designated plasmid pMP2L-M1 (Table S1). Similarly, another fragment carrying a 14-nucleotide substitution (5′-GTATGGATGCCAGTC-3′ to 5′-ACAATCAACATTAGTA-3′) in the region of the stem-loop structure and comparable in size (–344 to +228) was synthesised and recombined into an *Eco*RI/*Pst*I-cut pME6015 vector, which gave the plasmid pMP2L-M2 (Table S1).

### Quantitative Reverse Transcriptase-PCR (qRT-PCR)

Total RNA was extracted from the different target strains (M18 and M18ΔRA) and used as a template for qRT-PCR. Experiments were done according to the manufacturers’ instructions and the methods of Li *et al*. [16]. Two primer pairs, QRT-P2F/R and RPOD1/2, were designed based on the *phz2* and *rpoD* sequences in *P. aeruginosa* M18. PCRs were run using the following programme: one step at 95°C for 10 min and 40 cycles of 95°C for 15 s, 60°C for 20 s, and 72°C for 30 s. PCR analyses for each strain were repeated three times and data analysis was as described previously [19].

### Quantification of PCA Production

Extraction and quantification of PCA from a culture suspension was done as previously described [37]. For the present study, each experiment was done in triplicate.

### Assay for β-galactosidase (β-gal) Activity

*P. aeruginosa* M18 and its derivative strains that harboured the different fusion plasmids were cultured at 28°C with shaking at 200 rpm in a 500-mL Erlenmeyer flask containing 100 mL of PPM broth. Samples were collected at different time points, and the β-gal activity was assayed according to the methods of Miller [59] and as described by Huang *et al*. [19].

### Expression and Purification of Histidine-tagged RsmA from *E. coli*

A PCR product containing the 183-bp *rsmA* ORF was amplified by primers (PAEF/PAER) (Table S2) and subsequently cloned into *Nde*I-*Xho*I-cut T7-expression vector pET24a to generate a recombinant expression plasmid, pET24a-RsmA (Table S1), which was confirmed by sequencing and then transformed into *E. coli* BL21. C’ terminally histidine-tagged RsmA was overproduced by isopropyl-β-D-1-thiogalactopyranoside (IPTG) induction and purified as previously described [21]. After removing imidazole by high-performance liquid chromatography (HPLC) (AKTA, Hayward, CA), the final RsmA protein was stored at –70°C in buffer that contained 200 mM NaCl, 20 mM Tris-HCl (pH 8.0), 1 mM EDTA, and 1 mM DTT.

### In vitro Synthesis of the Unlabeled and Biotin-labeled RNAs

The *phz2* RNAs for the electrophoretic mobility shift assays (EMSA) were synthesized and/or labelled at their 3′ ends with biotin by TaKaRa Biotechnology Co., Ltd. (Dalian, China), and purified by anion exchange HPLC. Five RNA fragments are the *phz2-S1* (nt +50 to +89 relative to +1), *phz2-S2* (nt +92 to +131 relative to +1), *phz2-S3* (nt +150 to +180 relative to +1), *phz2-S4* (nt +175 to +205 relative to +1), and *phz2-S3M* (carrying a 3-nt substitution) mRNA 5′-UTRs.

### EMSA for RsmA–RNAs Interactions

EMSA was done using a Thermo Scientific LightShift Chemiluminescent RNA EMSA Kit according to the manufacturer’s instructions. RNA binding reaction mixtures (20 µL) included labelled RNA probes (1 nM), unlabelled RNA competitors (100 nM or 500 nM) and the target protein RsmA with increasing concentrations (0–400 nM) in the binding buffer (200 mM KCl, 100 mM HEPES, pH 7.3, 10 mM MgCl_2_, 2 µg tRNA, 5% glycerol, and 10 mM DTT). The reaction mixtures were incubated at room temperature for 30 min, mixed with 5 µL of loading buffer (0.1% bromophenol blue) and loaded on a 6% native polyacrylamide gel using TBE as the running dye. The resulting gels were scanned and visualised using the procedures of Wang *et al*. [21].

### Surface Plasmon Resonance

The Biacore T100 system and the streptavidin-coated sensor chip (SA chip, Biacore) were used for the kinetic experiment. A total of 120 resonance units (RUs) of biotin-labelled RNA (*phz2-S3*) were immobilized on the SA chip in the running buffer (200 mM NaCl, 20 mM Tris-HCl, PH 8.0, 1 mM EDTA, and 1 mM DTT) at flow rate of 10 uL min^−1^ for 60 s. The base line of resonance intensity was then allowed to stabilize for at least 60 min.

All the binding assays were performed at 25°C. The purified RsmA protein was diluted in the binding buffer to give a final concentration of 0, 20, 40, 80, 160, 320 or 640 nM, calculated for the RsmA protein, and then injected at the 30 µL min^−1^ constant flow rate. Injection of RsmA protein was terminated when the peak of fall line occurred. Dissociation of RsmA from RNA was monitored thereafter. The non-binding protein was washed out from the specific RNA-protein by the flow of binding buffer for an additional 200 s. Bound RsmA protein was removed from the complex by a solution of 2.0 M NaCl for 15 s at a flow rate of 30 µL min^−1^. To correct a nonspecific binding, the response from the reference protein was subtracted from the flow buffer (blank). Data were evaluated by the Biocore T100 evaluation software and the equilibrium binding constant (K_D_) was calculated for RsmA-RNA complex formation according to a previously described binding equation [60].

## Supporting Information

Figure S1 **Temperature-sensitive expression of the two** ***phz*** **gene clusters.** The PCA production at 37°C was measured and compared between the strains of M18ΔMSP1/M18ΔMSAP1 and M18ΔMSP2/M18ΔMSAP2 to detect the relative expression of each *phz* cluster affected by RsmA protein and temperature. Values are the mean ± standard deviation of triplicate cultures.
(TIF)

Table S1 **Strains and plasmids used in the present study.**
(DOC)

Table S2 **Primers used in the present study.**
(DOC)
