# Supplementary figures and images for: The phzA2-G2 Transcript Exhibits Direct RsmA-Mediated Activation in Pseudomonas aeruginosa M18
Source: PLoS One. 2014 Feb 24;9(2):e89653. doi: 10.1371/journal.pone.0089653 (PMC3933668; doi:10.1371/journal.pone.0089653)

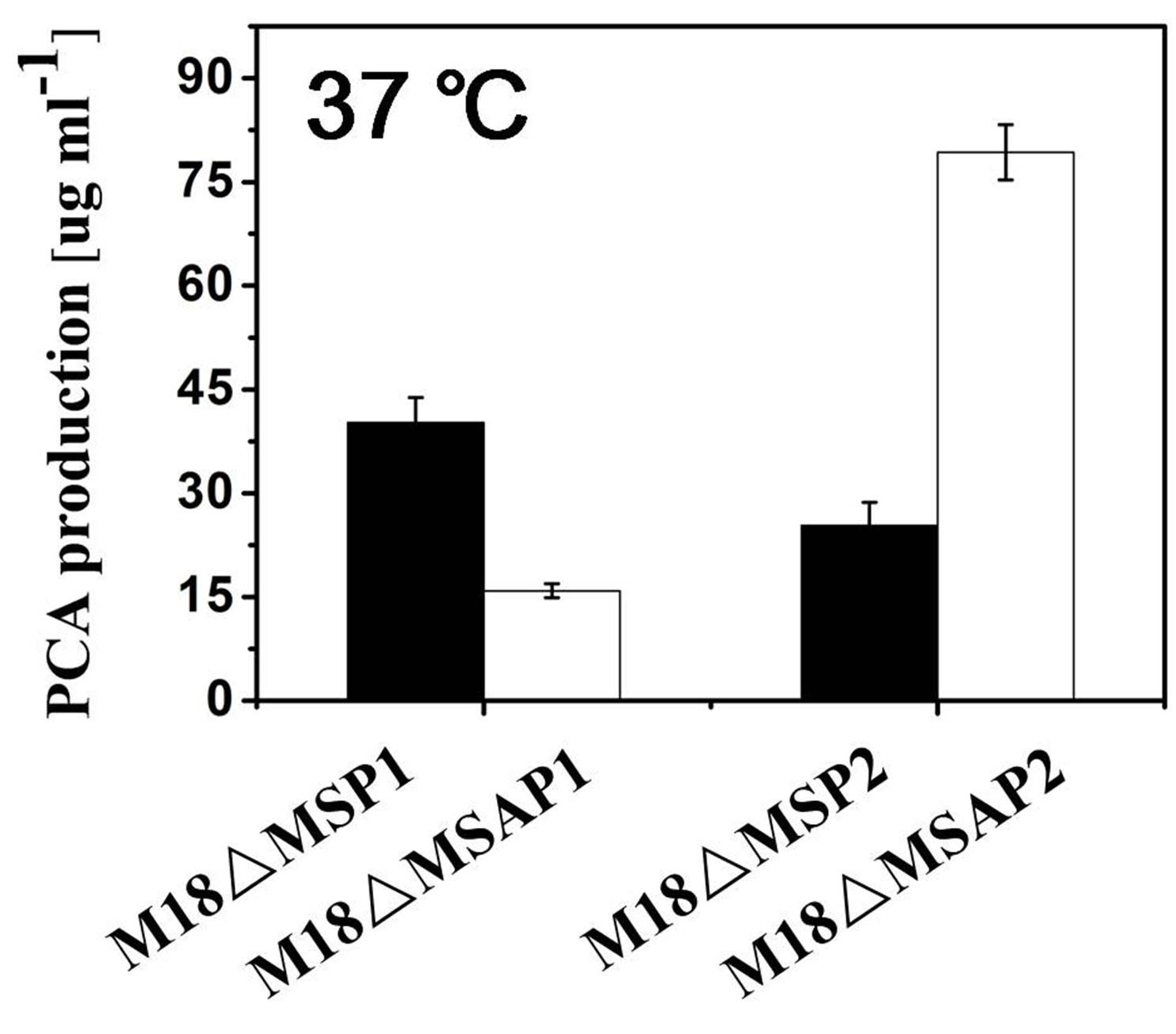

Supplement: Figure S1 — Temperature-sensitive expression of the two phz gene clusters. The PCA production at 37°C was measured and compared between the strains of M18ΔMSP1/M18ΔMSAP1 and M18ΔMSP2/M18ΔMSAP2 to detect the relative expression of each phz cluster affected by RsmA protein and temperature. Values are the mean ± standard deviation of triplicate cultures. (TIF) [file pone.0089653.s001.tif]
