# Supplementary material for: The phzA2-G2 Transcript Exhibits Direct RsmA-Mediated Activation in Pseudomonas aeruginosa M18
Source: PLoS One. 2014 Feb 24;9(2):e89653. doi: 10.1371/journal.pone.0089653 (PMC3933668; doi:10.1371/journal.pone.0089653)
Supplement: Table S1 — Strains and plasmids used in the present study. (DOC) [file pone.0089653.s002.doc]

**Table S1. Strains and plasmids used in the present study**

| **Strains/plasmids** | **Genotype and relevant characteristics** | **Reference** |
| --- | --- | --- |
| ***P. aeruginosa*** |  |  |
| M18 | Wild type, Sp^r^ | [1] |
| M18G | M18*gacA*::Km^r^, Sp^r^ Km^r^ | [2] |
| M18RYZ | M18*rsmY*::Gm^r^, M18*rsmZ*::Km^r^, Sp^r^ Gm^r^ Km^r^ | Present study |
| M18△RA | M18△*rsmA*, Sp^r^ | Present study |
| M18△MSP1 | M18△*phzM*△*phzS*△*phzA1-G1*, Sp^r^ | Present study |
| M18△MSAP1 | M18△*phzM*△*phzS*△*rsmA*△*phzA1-G1*, Sp^r^ | Present study |
| M18△MSP2 | M18△*phzM*△*phzS*△*phzA2-G2*, Sp^r^ | Present study |
| M18△MSAP2 | M18△*phzM*△*phzS*△*rsmA*△*phzA2-G2*, Sp^r^ | Present study |
| ***E. coli*** |  |  |
| S17 | TpR SmR *recA*, *thi*, *pro*, *hsdR*-M+RP4: 2-Tc:Mu: Km Tn7 λpir | [3] |
| BL21 (DE3) | *E.coli B,* F^-^ *, ompT, hsdSB(rB^-^mB^-^), gal, dcm* (DE3) | [4] |
| SM10 | thi-1 thr leu tonA lacY supE recA::RP4-2-Tc::Mu Km^r^ | [5] |
| DH5α | *supE*44 Δ*lac*U169(Φ80 *lacZ*ΔM15) *hsdR*17 *recA*1 *endA*1 *gyrA*96 *thi*-1 *relA*1 | [5] |
| **Plasmids** |  |  |
| pME6000 | Cloning vector, broad host range, Tc^r^ | Dieter Haas |
| p-*rsmA* | A 377 bp *EcoR*I-*Hind*III fragment containing the entire *rsmA* ORF and its operator region was cloned into pME6000, Tc^r^ | Present study |
| pEX18Tc | Gene replacement vector with MCS from pUC18, oriT+, sacB+, Tc^r^ | [5] |
| pUCGm | Source of Gm^r^ cassette, Gm^r^ | Dieter Haas |
| pUCKm | Source of Km^r^ cassette, Km^r^ | [5] |
| pEX-Y1Y2 | pEX18Tc containing a 836 bp *Sac*I-*Bam*HI upstream fragment and a 1005 bp *Bam*HI-*Hind*III downstream fragment flanking the *rsmY* gene, Tc^r^ | Present study |
| pEX-Y1Y2-Gm | A 825 bp *Bam*HI-*Bam*HI Gm^r^ fragment was cloned into pEX-Y1Y2, Tc^r^ Gm^r^ | Present study |
| pEX-Z1Z2 | pEX18Tc containing a 913 bp *Hind*III-*Xba*I upstream fragment and a 898 bp *Xba*I-*Kpn*I downstream fragment flanking the *rsmZ* gene, Tc^r^ | Present study |
| pEX-Z1Z2-Km | A 1250 bp *Xba*I-*Xba*I Km^r^ fragment was cloned into pEX-Z1Z2, Tc^r^ Km^r^ | Present study |
| pK18mobsacB | Allelic exchange vector, *sacB*^+^, Kan^r^ | [6] |
| pK18-A1A2 | pK18mobsacB containing a 1052 bp *Eco*RI-*Hind*III fragment with a 400 bp deletion of *rsmA* gene, Km^r^ | Present study |
| pK18-P1P2 | pK18mobsacB containing a 1904 bp *BamH*I-*Xbal*I fragment with a 6238 bp deletion of *phzA1-G1* gene cluster, Km^r^ | Present study |
| pK18-P3P4 | pK18mobsacB containing a 2292 bp *BamH*I-*Xbal*I fragment with a 6221 bp deletion of *phzA2-G2* gene cluster, Km^r^ | Present study |
| pET24a | T7 expression vector, Km^r^ | Geng Wu |
| pET24a-RsmA | A 192 bp *Nde*I-*Xho*I fragment containing the 183 bp *rsmA* ORF of *P. aeruginosa* M18 was cloned into pET24a, Km^r^ | Present study |
| pME6015 | pVS1-p15A shuttle vector for constructing the translational *lacZ* fusions, Tc^r^ | [7] |
| pME6522 | pVS1-p15A shuttle vector for constructing the transcriptional *lacZ* fusions, Tc^r^ | [8] |
| pMP1C | A transcriptional *phzA1-lacZ* fusion containing a 341 bp fragment upstream of the *phzA1* transcriptional start site (TSS) in pME6522, Tc^r^ | [9] |
| pMP2C-2 | A transcriptional *phzA2-lacZ* fusion containing a 210 bp fragment upstream of the *phzA2* transcriptional start site (TSS) in pME6522, Tc^r^ | Present study |
| pMP1L | A translational *phzA1′-′lacZ* fusion containing a 692 bp of the 5´-UTR and the first 9 codons of *phzA1* in pME6015, Tc^r^ | [9] |
| pMP2L | A translational *phzA2′-′lacZ* fusion containing a 544 bp of the 5´-UTR and the first 9 codons of *phzA2* in pME6015, Tc^r^ | [9] |
| pMP2L-M1 | A replaced *phzA2′-′lacZ* fusion containing 7 bp substitution in the RsmA target motif in pMP2L, Tc^r^ the *phz1* TSS in pME6522, Tc^r^ | Present study |
| pMP2L-M2 | A replaced phzA2′-′lacZ fusion containing 14 bp substitution in the stem-loop region covering RsmA target motif in pMP2L, Tc^r^ | Present study |
| p9533-phz2o-6 | A post-transcriptional phz2o-6-lacZ operator fusion containing a 228 bp fragment downstream of the phz2 TSS in pME9533, Tc^r^ | [4] |
| p9533-phz2-D1 | A post-transcriptional lacZ fusion containing a 135 bp fragment covering from +94 to +228 downstream of the phz2 TSS in pME9533, Tc^r^ | Present study |
| p9533-phz2-D2 | A post-transcriptional lacZ fusion containing a 107 bp fragment covering from +122 to +228 downstream of the phz2 TSS in pME9533, Tc^r^ | Present study |

**Reference**

1. Huang X, Zhu D, Ge Y, Hu H, Zhang X, et al. (2004) Identification and characterization of pltZ, a gene involved in the repression of pyoluteorin biosynthesis in Pseudomonas sp. M18. FEMS Microbiol Lett 232: 197-202.
2. Ge Y, Huang X, Wang S, Zhang X, and Xu Y (2004) Phenazine-1-carboxylic acid is negatively regulated and pyoluteorin positively regulated by gacA in Pseudomonas sp. M18. FEMS Microbiol Lett 237: 41-47.
3. Schafer A, Kalinowski J, Simon R, Seep-Feldhaus AH, Puhler A (1990) High-frequency conjugal plasmid transfer from gram-negative Escherichia coli to various gram-positive coryneform bacteria. J Bacteriol 172: 1663-1666.
4. Wang G, Huang X, Li S, Huang J, Wei X, et al. (2012) The RNA chaperone Hfq regulates antibiotic biosynthesis in the rhizobacterium Pseudomonas aeruginosa M18. J Bacteriol 194: 2443-2457.
5. Huang J, Xu Y, Zhang H, Li Y, Huang X, et al. (2009) Temperature-dependent expression of phzM and its regulatory genes lasI and ptsP in rhizosphere isolate Pseudomonas sp. strain M18. Appl Environ Microbiol 75: 6568-6580.
6. Schafer A, Tauch A, Jager W, Kalinowski J, Thierbach G, et al. (1994) Small mobilizable multi-purpose cloning vectors derived from the Escherichia coli plasmids pK18 and pK19: selection of defined deletions in the chromosome of Corynebacterium glutamicum. Gene 145: 69-73.
7. Heeb S, Blumer C, Haas D (2002) Regulatory RNA as mediator in GacA/RsmA-dependent global control of exoproduct formation in Pseudomonas fluorescens CHA0. J Bacteriol 184: 1046-1056.
8. Blumer C, Heeb S, Pessi G, Haas D (1999) Global GacA-steered control of cyanide and exoprotease production in Pseudomonas fluorescens involves specific ribosome binding sites. Proc Natl Acad Sci U S A 96: 14073-14078.
9. Li Y, Du X, Lu ZJ, Wu D, Zhao Y, et al. (2011) Regulatory feedback loop of two phz gene clusters through 5'-untranslated regions in Pseudomonas sp. M18. PLoS One 6: e19413.
