# Supplementary material for: The phzA2-G2 Transcript Exhibits Direct RsmA-Mediated Activation in Pseudomonas aeruginosa M18
Source: PLoS One. 2014 Feb 24;9(2):e89653. doi: 10.1371/journal.pone.0089653 (PMC3933668; doi:10.1371/journal.pone.0089653)
Supplement: Table S2 — Primers used in the present study. (DOC) [file pone.0089653.s003.doc]

**Table S2. Primers used in the present** study

| **Primer^a^** | **Nucleotide sequence** | **Restriction site^b^** |
| --- | --- | --- |
| PY1F | 5´- ATAT*GAGCTC*CGAGGTGGATGATGCC-3´ | *Sac* I |
| PY1R | 5´- CGAT*GGATCC*ACGGTTTGAAGATTACGC-3´ | *BamH* I |
| PY2F | 5´- ATAC*GGATCC*ACGGATGTCAGGATAGAGG -3´ | *BamH* I |
| PY2R | 5´- CTAT*AAGCTT*CGGGACCCACTCCATTT -3´ | *Hind* III |
| PZ1F | 5´- CTAT*AAGCTT*CTGGTCGGAAGCGGATGATC -3´ | *Hind* III |
| PZ1R | 5´- CGC*TCTAGA*CTTCGGCAGCCATTTTAG -3´ | *Xba* I |
| PZ2F | 5´- CGC*TCTAGA*TGACGAGGGACTGAAGAG -3´ | *Xba* I |
| PZ2R | 5´- TAT*GGTACC*GTTCGCCACCCACTACTTC -3´ | *Kpn* I |
| PA1F | 5´- CG*GAATTC*GCCTCGGTTCCAAGGTGCT -3´ | *EcoR* I |
| PA1R | 5´- CAGCATTCCTTTCTCCTCACGC -3´ |  |
| PA2F | 5´- GAGAAAGGAATGCTGGAGAAAGATCAAGAGCCAAACCAT -3´ |  |
| PA2R | 5´- CCC*AAGCTT*CTTCAACAGTGACGAGCATCCG -3´ | *Hind* III |
| PP1F | 5´- AGAA*GGATCC*AGAACAGCACCATGTC -3´ | *BamH* I |
| PP1R | 5´- TTTTAA*GAGCTC*CCCTGTACCGCTGA-3´ | *Sac* I |
| PP2F | 5´- ATTA*GAGCTC*CACCGCTACCTGCAAC -3´ | *Sac* I |
| PP2R | 5´- TCGG*TCTAGA*GGCGACGATAATGGTC -3´ | *Xba* I |
| PP3F | 5´- AAAT*GGATCC*GCTGATCTGGAATGGC -3´ | *BamH* I |
| PP3R | 5´- TTAT*GAGCTC*CCCTTTCAACCGTTGG -3´ | *Sac* I |
| PP4F | 5´- ATAT*GAGCTC*TGGAAGCACCGCTACC -3´ | *Sac* I |
| PP4R | 5´- CGCG*TCTAGA*GGGTTTCTTCGATCAC -3´ | *Xba* I |
| PACF | 5´- GCC*GAATTC*TTTGACCGTTTGGCAGGAA -3´ | *EcoR* I |
| PACR | 5´- CCC*AAGCTT*GCATGATACCCATCTTTACCC -3´ | *Hind* III |
| PAEF | 5´- CAGT*CATATG*CTGATTCTGACTCGT -3´ | *Nde*I |
| PAER | 5´- CAA*CTCGAG*ATGGTTTGGCTCTTGATC -3´ | *Xho*I |
| P2CF | 5´- TATAT*GAATTC*GACGCCTCGTCGCCTAGC -3´ | *EcoR* I |
| P2CR | 5´- TATA*CTGCAG*GACAGGGGACAAACTTATAAACGCT -3´ | *Pst* I |
| QRT-P2F | 5´- TCCCCTGTCAAATCTGGTTA -3´ |  |
| QRT-P2R | 5´- CGACTGGCATCCATACCG -3´ |  |
| RPOD1 | 5´- GAGCGGGAGGAGCGTTTAC -3´ |  |
| RPOD2 | 5´- CGGGCAAAAAATAAGCAGAGG -3´ |  |

^a^ All primers were obtained from Invitrogen (Shanghai, China).

^b^ The restriction sites used in the cloning procedure are underlined.
